# Synthesis of the dumbbell AuNRs@BCP nanoparticles *via* UV light-initiated RAFT polymerization-induced self-assembly

**DOI:** 10.1039/d5ra06140g

**Published:** 2026-01-29

**Authors:** Jian Wang, Feng Xu, Zhenzhong Liu, Linli Xu, Mingyue Liu, Ye Deng, Yue Sun, Yanjing Zhang, Dan-Hua Wang, Youju Huang

**Affiliations:** a Chemical Pharmaceutical Research Institute, College of Medicine and Pharmaceutical Engineering, Taizhou Vocational and Technical College Taizhou 318000 China; b Taizhou Key Laboratory of Medical Devices and Advanced Materials, Taizhou Institute of Zhejiang University Taizhou 318000 China zzliu@zju.edu.cn; c School of Pharmaceutical and Chemical Engineering, Taizhou University Taizhou 318000 China liumingyue0820@126.com; d College of Materials, Chemistry and Chemical Engineering, Hangzhou Normal University Hangzhou 311121 China yjhuang@hznu.edu.cn

## Abstract

Herein, we report a novel method to synthesize the dumbbell AuNRs@BCP nanoparticles *via* UV light-initiated RAFT polymerization-induced self-assembly *in situ*. This strategy provides a promising alternative for synthesizing asymmetric inorganic/polymeric hybrid nanoparticles with tunable morphologies. Shape-dependent surface-enhanced Raman scattering (SERS) experiments in dilute dispersions, along with electric field simulations, were conducted. Due to the plasmonic hotspots formed between the AuNRs and BCP domains, the dumbbell AuNRs@BCP nanoparticles were ideal for generating strong and reproducible SERS signals for methyl orange.

## Abstract 2

Novel dumbbell AuNRs@BCP nanoparticles were explored *via* the UV light-initiated RAFT PISA method.

## Graphical abstract

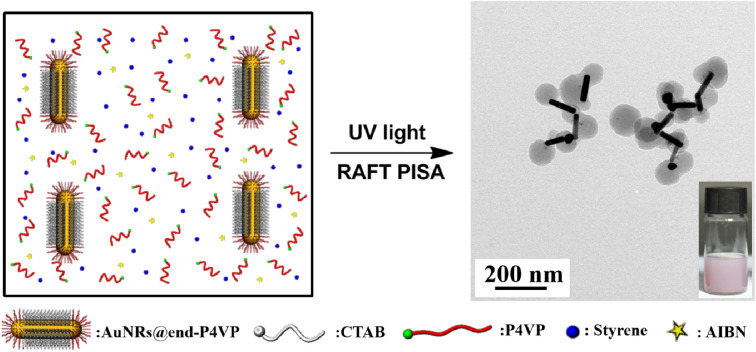

## Introduction

Inorganic/organic hybrid nanoparticles^1,2^ are among the most promising nanomaterials due to their potential applications in various fields such as sensing,^3^ nanomedicine,^4^ and catalysis.^5^ Because both their architecture and molecular weight can be precisely designed and adjusted, block copolymer (BCP) self-assembly^6,7^ is a powerful strategy for fabricating diverse inorganic/organic nanoparticles. Especially, gold nanoparticles (AuNPs)^8,9^ can be selectively encapsulated within different BCP morphologies (*e.g.* micelles,^10^ rod-shaped micelles,^11^ and vesicles^12^) through specific self-assembly techniques. However, these nanohybrids are typically prepared from spherical gold nanoparticles *via* non-*in situ* encapsulation methods.

Unlike spherical nanoparticles, non-spherical nanoparticles such as nanotubes,^13^ nanowires^14^ and nanorods,^15^ exhibit unique anisotropic properties in heat, light, electricity, and magnetism due to their additional orientational entropy. Gold nanorods (AuNRs) are rod-shaped nanoparticles whose surface plasmon resonance properties can be tuned by adjusting their aspect ratio.^16,17^ Furthermore, hybridizing AuNRs with inorganic materials can readily produce full-patched, dumbbell, and side-patched structures.^18–20^ For example, Adelt *et al.* systematically investigated the anisotropic coating of AuNRs ends with silica and examined how growth conditions influence silica shell formation.^21^ Lee *et al.* reported a novel method for synthesizing polymer-free side-silica-patched gold nanorod structures with exposed hotspots without using polymeric ligands.^22^ Pons *et al.* synthesized dumbbell AuNRs@TiO_2_ to produce hydroxyl radical species under near-infrared light (NIR) irradiation, demonstrating that the photodynamic effect induces cell death more efficiently than the photothermal effect alone.^23^

Compared to inorganic materials, incorporating AuNRs into block copolymers leads to enhanced mechanical and optical properties.^24,25^ For example, Li *et al.* reported that AuNRs tethered to polystyrene (PS) with bimodal chain lengths can be effectively incorporated into the cylindrical PS domains of poly(4-vinylpyridine)-*b*-polystyrene (P4VP-*b*-PS) through a directed supramolecular assembly.^26^ Grzelczak *et al.* achieved the formation of core–shell hybrid nanomaterials by encapsulating AuNRs within the PS domain of polystyrene-*b*-poly(acrylic acid) (PS-*b*-PAA) micelles.^27^ Recently, several pioneering studies have been developed to achieve asymmetric AuNRs@BCP nanostructures. For example, Wang *et al.* reported simple heat-induced transformations of PS-*b*-PAA shells in various modes (contraction, bimodal contraction, dissociation, and winding), fabricated through a general method involving AuNRs encapsulation with different ligands and concentrations during the initial encapsulation step.^28^ Moreover, it is still a great challenge to fabricate AuNRs@BCP nanoparticles with precisely controlled nanostructures at high yields.

Over the past decade, polymerization-induced self-assembly (PISA)^29,30^ has emerged as an efficient one-pot method for synthesizing various highly concentrated block copolymer nanoparticles, as well as inorganic/polymeric hybrid nanoparticles, across diverse solvent systems. Due to its compatibility with a wide range of monomers, solvents, and reaction conditions, reversible addition-fragmentation chain transfer (RAFT) polymerization^31^ remains a robust PISA technique. In addition to thermal initiation, the PISA process can also be carried out through other stimuli, such as light irradiation.^32^ For example, we reported a UV-light-induced RAFT PISA method conducted under mild conditions to prepare polymeric micelles with high solid contents at room temperature.^33^ Up to now, various hybrid nanomaterials incorporating organic or inorganic components have been constructed *via* the PISA method.^34^ Hou *et al.* utilized two macro-CTAs, one grafted onto silica particles and the other dissolved in solution, to fabricate surface micelles with different morphologies and sizes on silica particles.^35^ We fabricated the Janus AuNPs@P4VP-*b*-PS nanoparticles *via* UV light-initiated RAFT PISA, which can be precisely tuned by adjusting the reaction conditions and polymerization time.^36^ However, there have been no reports on the hybridization of AuNRs with BCP self-assembly during the polymerization process till now.

## Experimental

### Materials

Chloroauric acid tetrahydrate (HAuCl_4_·4H_2_O), silver nitrate (AgNO_3_), cetyltrimethylammonium bromide (CTAB), sodium oleate (NaOL), ascorbic acid (AA), sodium borohydride (NaBH_4_), hydrochloric acid (HCl), methanol, alumina (Al_2_O_3_), and calcium hydride (CaH_2_) were purchased from Sigma-Aldrich. Methyl orange (MO) was obtained from Aladdin. 4-Vinylpyridine (4VP) was dried using CaH_2_ and then purified through vacuum distillation. The styrene monomer was purified using an alumina column. 2,2′-Azodiisobutyronitrile (AIBN) was recrystallized from methanol and stored at 0 °C. P4VP-CTA with Mn = 4500 g mol^−1^ was synthesized *via* the RAFT polymerization method^33^ and confirmed by ^1^H NMR analysis. The UV light source is a UV nail gel curing lamp (*λ*_max_ = 365 nm) equipped with 4 × 9 W bulbs. The light intensity was measured as 2.50 mW cm^−2^ using a UV radiometer.

### Synthesis of gold nanorods (AuNRs)

The AuNRs were prepared using the seed growth method,^37^ which involved two steps: the synthesis of crystal seeds and the formulation of the growth solution. (1) First, HAuCl_4_ (5.0 mL, 0.5 mM) and CTAB (5 mL, 0.2 M) were added to a 20 mL glass bottle. Subsequently, a freshly prepared NaBH_4_ solution (0.6 mL, 0.01 M), diluted with 1.0 mL of H_2_O, was rapidly introduced into the mixture, causing the solution color to change from yellow to light brown. The residual NaBH_4_ was completely hydrolyzed by curing at room temperature for 30 min. (2) Next, 7.0 g of CTAB and 1.234 g of NaOL were dissolved in 250 mL of H_2_O at 60 °C. The solution was then cooled to room temperature before adding AgNO_3_ (18 mL, 4 mM) and kept for 15 min. Subsequently, HAuCl_4_ (250 mL, 1 mM) was added to the solution and stirred at 700 rpm for 90 min. Then, hydrochloric acid (2.1 mL, 12.1 M) was added to adjust the pH, and the solution was further stirred at 400 rpm for about 15 min. Afterwards, AA (1.25 mL, 0.064 M) was added with vigorous stirring for 30 seconds. Finally, 0.8 mL of the seed solution was added and stirred vigorously for 30 seconds, then left to stand for 12 h at room temperature.

### Modification of polymer-decorated AuNRs@P4VP

The P4VP-CTA was selectively modified on the surface of AuNRs following the method reported in the relevant literature.^38^ The main experimental steps are as follows: First, 3.7 mL of AuNRs stock solution was centrifuged at 7000 rpm for 30 min to remove excess CTAB. The supernatant was then discarded, and the precipitate at the bottom was collected and dispersed in a methanol/water (1 : 1) solution. Under ultrasonic conditions, a certain amount of P4VP dissolved in methanol/water (1 : 1) was added to the concentrated AuNRs. Finally, it was placed at room temperature for 24 h. After three centrifugation cycles (7500 rpm for 30 min) and dispersion treatment, excessive polymers and other impurities were removed. Finally, the volume is set to 500 µL and stored in the refrigerator before use.

By fixing AuNRs and adjusting the amount of P4VP, AuNRs can be selectively modified. The general modification process is to replace AuNRs with 0.05% P4VP in methanol/water = 1 : 1 to obtain polymer-modified AuNRs@P4VP. The effect of P4VP dosage on the UV-Vis absorption peak of AuNRs was observed by UV-Vis absorption spectroscopy.

In order to verify the photostability of polymer-modified AuNRs@P4VP under UV irradiation, the AuNRs@P4VP solution was irradiated under UV light for different times and UV-Vis absorption spectra were recorded.

### Preparation of the dumbbell AuNRs@BCP nanoparticles

The dumbbell AuNRs@BCP nanoparticles were prepared using a UV light-initiated RAFT PISA method.^36^ Herein, AuNRs@end-P4VP was used as the seed, P4VP as the chain transfer agent, styrene as the monomer, and AIBN as the photoinitiator, which were added to methanol. After three cycles of “freezing-vacuum-thawing”, the bottle was sealed and irradiated under UV light. As a specific example, AuNRs@end-P4VP (750 L), P4VP (4 mg, 4.1 × 10^−4^ mmol), styrene (0.07 g, 0.70 mmol), and AIBN (0.02 mg, 1.2 × 10^−4^ mmol) were added to 3.75 mL of methanol in a glass bottle, which was then irradiated and polymerized under UV light for 18 h.

### Surface-enhanced Raman scattering (SERS) measurements

Raman and SERS spectra were recorded using a Raman spectrometer under 785 nm irradiation.^39^ The dispersions were placed in 8 mm vials for measurement. Raman and SERS measurements with or without the addition of MO molecules into the sample were conducted in dilute dispersions without any aggregation. The final concentration of MO in each dispersed sample was on the order of 10^−3^ to 10^−8^ M in the water medium. The acquisition time was 10 s, and all spectra were obtained for three cycles. Spectra were recorded in the range of 500–2000 cm^−1^ with a resolution of about 10 cm^−1^.

### Electric field simulations of the dumbbell AuNRs@BCP nanoparticles

The simulations employed the finite element method (FEM) to analyze local field distributions on the surfaces of AuNRs with polymer coatings oriented in different directions. The 3D model was constructed based on experimental TEM data. The frequency domain in the wave optics module was employed to simulate the electric field distribution, with a fixed light wavelength of 785 nm, polarized in the *x*-direction and incident along the *z*-direction. The refractive index of air at this wavelength was set as 1, while those of Au, P4VP, and PS were taken from the reference values.^40–42^

### Characterizations

The microstructure of the nanoparticles was observed using a Tecnai F20 TEM operated at 200 kV. The sample preparation involved centrifugation, dilution, and dispersion in methanol. A portion of this solution was then applied to a copper grid supported by a 300-mesh carbon film. TEM analysis was performed after the solvent had evaporated. Fourier transform infrared spectroscopy (FT-IR) analysis was performed with a Nicolet 6700. The spectral scanning range was 400–4000 cm^−1^, and the sample was obtained by the KBr tablet method. Thermogravimetric (TG) analysis was conducted using the PerkinElmer Diamond under a nitrogen atmosphere, with a gas flow rate of 50 mL min^−1^, heating rate of 10 °C min^−1^, and temperature range of 50 °C to 800 °C. The diameter distribution and zeta potential of the samples were determined using a Malvern Laser Zeta meter equipped with a 633 nm He–Ne laser. At least three measurements of each sample were taken to check for result repeatability. The proton nuclear magnetic resonance (^1^H NMR) spectra were obtained on a 400 MHz NMR instrument using CDCl_3_ as the solvent and with tetramethylsilane (TMS) as an internal standard. The UV-Vis absorption spectra were conducted on a PerkinElmer Lambda 950 over the wavelength range from 400 nm to 1100 nm.

## Results and discussion

**Scheme 1:**
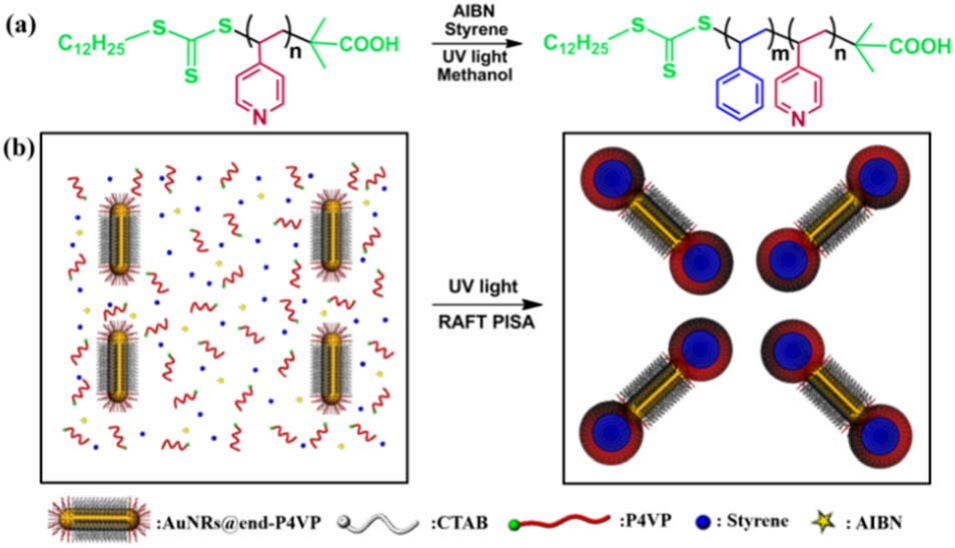
(a) Synthesis of the P4VP-*b*-PS block copolymers. (b) Fabrication of dumbbell AuNRs@BCP *via* UV light-initiated RAFT PISA.

Herein, a novel methodology utilizing the UV light-induced RAFT PISA method was conducted to prepare the dumbbell AuNRs@P4VP-*b*-PS nanoparticles. The preparation process is illustrated in Scheme 1. The P4VP chain transfer agent (CTA) was synthesized through thermal-initiated RAFT solution polymerization (Scheme 1a). The cetyltrimethylammonium bromide (CTAB)-capped AuNRs were prepared using the seed growth method. Transmission electron microscopy (TEM) image of the AuNRs is shown in Fig. S1a, revealing uniform morphologies with a diameter of 10 nm and a length of 100 nm. The UV-Vis spectrum indicated two absorption peaks at 510 nm and 910 nm in a methanol:H_2_O solution (Fig. S1b). Subsequently, CTAB-capped AuNRs were functionalized with P4VP-CTA to obtain polymer-tethered AuNRs@end-P4VP. These AuNRs@end-P4VP were then added into the reaction system as seeds. Upon UV light irradiation, styrene polymerizes to form PS chains, and the P4VP-*b*-PS block copolymer spontaneously self-assembles in methanol to form polymeric nanomicelles at both ends of the AuNRs@end-P4VP, resulting in the dumbbell AuNRs@BCP nanoparticles (Scheme 1b).

Initially, the experimental system with a mole ratio of P4VP/St/AIBN = 3 : 5700 : 1 and a specific amount of CTAB-capped AuNRs as seeds was investigated (Table S1). After three cycles of “freeze-vacuum-thawing” for deaeration treatment, it was sealed and irradiated with UV light for 18 h. The color of the solution changed from transparent to an opaque cloudy state. After washing with methanol, the centrifuged dispersant was characterized using TEM, which showed that there were both blank P4VP-*b*-PS micelles (Fig. S2a) and partially hybridized AuNRs with micelles (Fig. S2b) in the system. This may be attributed to the ligand exchange effect, which hindered the successful hybridization between CTAB-capped AuNRs and P4VP-*b*-PS micelles.^43^

**Fig. 1:**
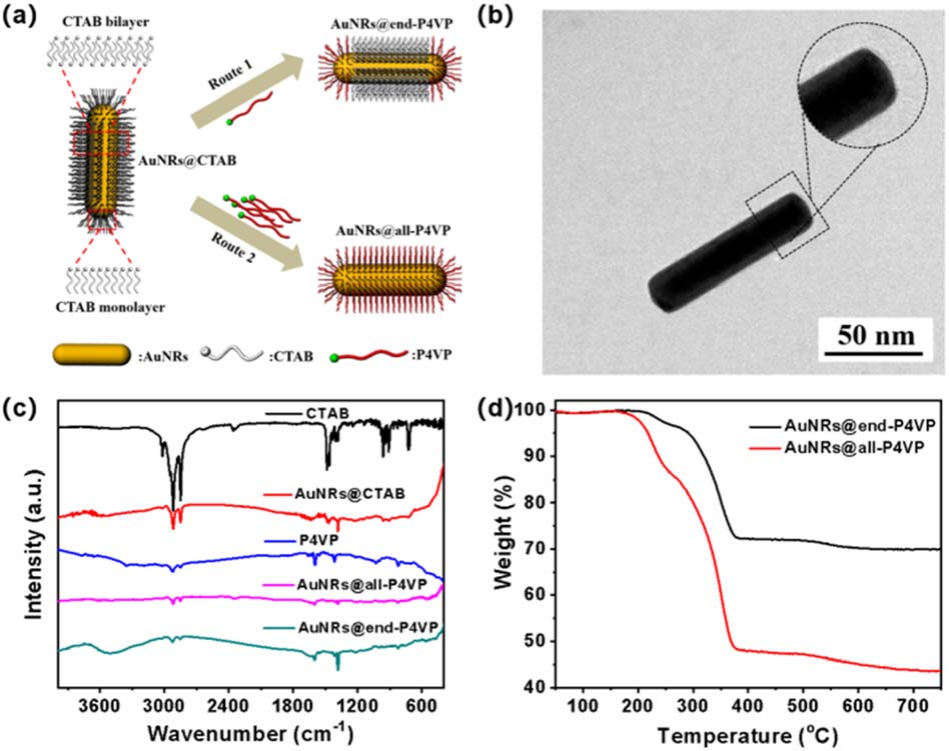
(a) P4VP-CTA substitution diagram of AuNRs@CATB to obtain AuNRs@end-P4VP and AuNRs@all-P4VP. (b) TEM images of AuNRs@end-P4VP. (c) FT-IR spectra of related substances and nanoparticles. (d) TG curves of AuNRs@end-P4VP and AuNRs@all-P4VP.

In order to eliminate the ligand exchange effect and improve the quality of hybridization, CTAB-capped AuNRs can be functionalized with polymer ligands,^44^ which help stabilize AuNRs in organic solvents. Due to the different curvature between the side and end sections of AuNRs, the surface adsorption energy of surfactants in these regions varies significantly. It has been reported that the adsorption of loosely bound CTAB on the end surfaces of AuNRs, forming a single molecular layer, while a dense CTAB bilayer is formed on the side surfaces.^45^ In theory, selective modification of AuNRs can be achieved by controlling the quantity of modified ligands added. According to the literature,^46^ the modification of AuNRs was conducted by tuning the ratios of AuNRs to P4VP-CTA (Fig. 1a): when a small amount of P4VP-CTA is used, only selectively end-polymer modified AuNRs (AuNRs@end-P4VP) are obtained (Path 1); conversely, when an excess of P4VP-CTA is used, fully modified AuNRs (AuNRs@all-P4VP) are acquired (Path 2). A measured amount of the P4VP-CTA solution was added to the concentrated AuNR solution and incubated at room temperature for 24 h. After the replacement, CTAB and excess P4VP-CTA were removed by centrifugation.

Firstly, the AuNRs modified with different amounts of P4VP were characterized using UV-Vis spectroscopy. The peak of AuNRs@CTAB at 910 nm gradually red-shifted to approximately 920 nm as the P4VP concentration increased (Fig. S3). This shift is attributed to changes in the chemical environment on the AuNRs surface.^47^ The TEM images of AuNRs@end-P4VP nanoparticles illustrated a polymer shell about 4 nm thick at the ends of the AuNRs (Fig. 1b), whereas AuNRs@all-P4VP nanoparticles displayed a polymer shell covering both the ends and the side surfaces (Fig. S4). The characteristic vibration peaks of CTAB, P4VP-CTA, and the surface ligands of AuNRs were identified through FT-IR analysis. As shown in Fig. 1c, CTAB exhibits C–H stretching vibrations at 2918 cm^−1^ and 2850 cm^−1^, along with methyl bending vibrations at 1487 cm^−1^ and 1473 cm^−1^. The P4VP-CTA displays a carboxyl stretching vibration at 3335 cm^−1^ and pyridine ring vibrations at 1660 cm^−1^, 1633 cm^−1^, and 1598 cm^−1^. The spectrum of AuNRs@end-P4VP confirms the coexistence of both CTAB and P4VP-CTA on the surface. Furthermore, both AuNRs@end-P4VP and AuNRs@all-P4VP were analyzed using TG analysis. As shown in Fig. 1d, AuNRs@end-P4VP exhibited two decomposition ranges: a 5% weight loss of side groups between 140–250 °C and a 25% loss of the polymer skeleton between 250–450 °C, resulting in a 70% residue. In comparison, AuNRs@all-P4VP showed a 15% and 40% losses, leaving a 45% residue. The decoration of P4VP-CTA was also characterized by zeta potential measurements. The results in Fig. S5 showed that P4VP-CTA had a peak at −5.99 mV, and the AuNRs@CTAB had a peak at 30.14 mV. After decoration with P4VP-CTA, the zeta potential of AuNRs@end-P4VP slightly decreased to 25.84 mV, indicating that the surface remains positively charged in distilled water. Finally, the stability of AuNRs@end-P4VP in solution under UV light irradiation was investigated. The UV-Vis spectral results in Fig. S6 indicated that the curves remained stable for 6 h.

**Fig. 2:**
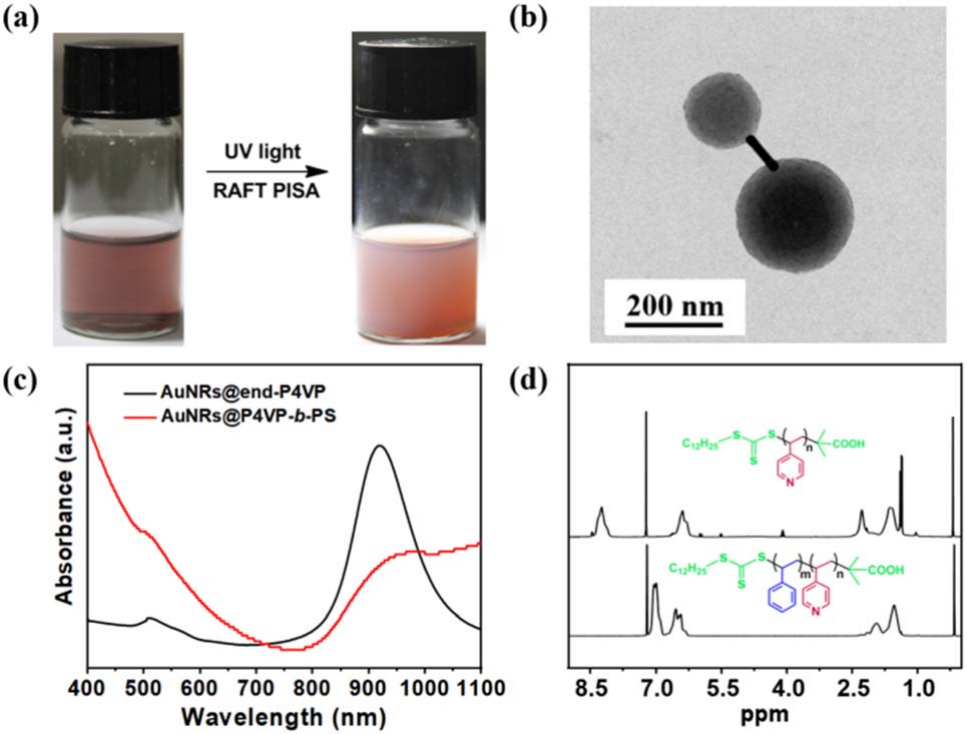
(a) Optical photographs showing the successful synthesis of dumbbell AuNRs@BCP: color change of the solution before and after UV light-initiated RAFT PISA. (b) TEM images of the dumbbell AuNRs@BCP; (c) UV-Vis spectra of AuNRs@end-P4VP and dumbbell AuNRs@BCP in methanol. (d) ^1^H NMR spectra of P4VP-CTA and the P4VP-*b*-PS block copolymers purified from dumbbell AuNRs@BCP.

Then, the experimental system with the same mole ratio of P4VP/St/AIBN and AuNRs@end-P4VP as the seed was conducted. After irradiation under UV light for 18 h, the transparent brown solution transformed into a completely opaque pink cloudy liquid (Fig. 2a). The resulting nanoparticles were purified and characterized by TEM. As shown in Fig. 2b, the ends of the AuNRs were embedded in the shell of P4VP-*b*-PS, resulting in a unique dumbbell morphology. The hydrodynamic radius of dumbbell AuNRs@P4VP-*b*-PS nanoparticles was also characterized by dynamic light scattering (DLS) analysis. As shown in Fig. S7, the diameter of the dumbbell AuNRs@BCP was 187.5 nm, whereas that of the AuNRs@end-P4VP was only 97.68 nm. Furthermore, the purified dumbbell AuNRs@P4VP-*b*-PS were characterized by UV-Vis spectroscopy, which exhibited two distinct absorption peaks at 510 nm and 960 nm, as shown in Fig. 2c. This observation is consistent with previous reports indicating that the localized surface plasmon resonance (LSPR) of AuNRs undergoes a red-shift after the dumbbell silica coating.^48^ The dumbbell AuNRs@BCP can be destroyed and dissolved in organic solvents. After centrifugation to remove the AuNRs precipitate, the supernatants were subsequently precipitated in *n*-hexane to yield white solids. The resulting polymers were characterized by ^1^H NMR spectroscopy. As illustrated in Fig. 2d, P4VP-CTA displayed distinct signals at *δ* = 8.31 and *δ* = 6.57 ppm (pyridine ring), while P4VP-*b*-PS only exhibited signals at *δ* = 6.57 and 7.09 ppm (phenyl ring).^33^ An additional experiment using AuNRs@all-P4VP as a seed was also conducted. The TEM results in Fig. S8 indicated that AuNRs could be embedded within the P4VP-*b*-PS micelles to form core–shell AuNRs@BCP.

**Fig. 3:**
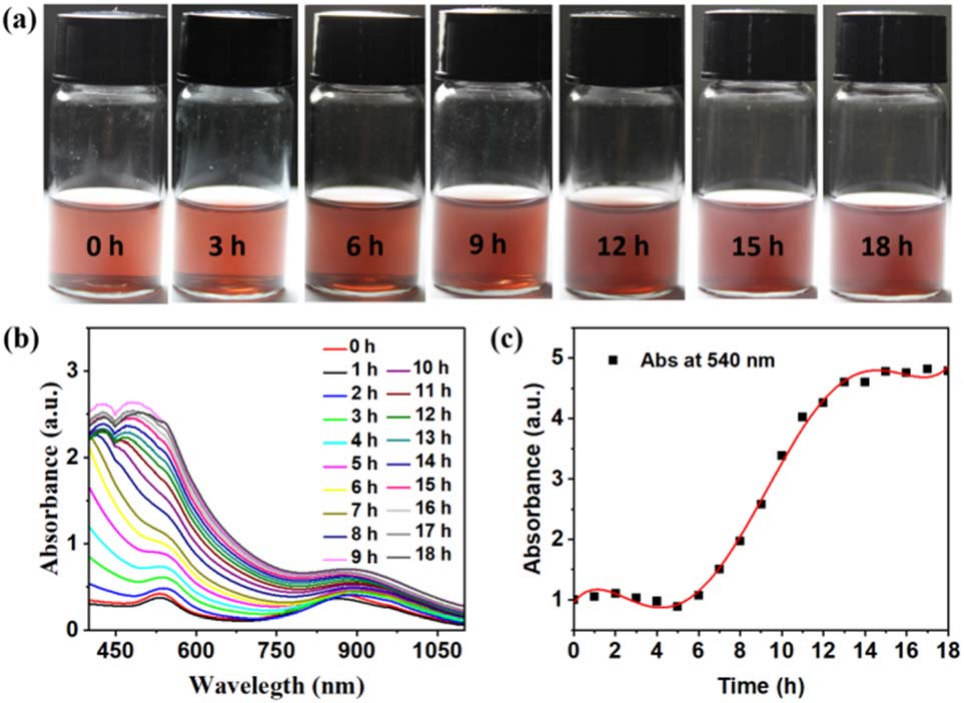
(a) Optical photographs of dumbbell AuNRs@BCP prepared by UV light-initiated RAFT PISA at various polymerization times. (b) Polymerization process monitored by UV-Vis spectroscopy. (c) Polymerization curves calculated by plotting the peak intensity at 540 nm *versus* polymerization times.

To further investigate the polymerization kinetics of the dumbbell AuNRs@BCP nanoparticles, the aforementioned system was irradiated with UV light for different times. Optical images of the reaction process were taken at 0 h, 3 h, 6 h, 9 h, 12 h, 15 h, and 18 h, respectively. As illustrated in Fig. 3a, the color of the solution showed no significant change during the 3 h of UV irradiation. Subsequently, the solution appeared cloudy at 6 h and ultimately turned completely opaque by 18 h. The process was also monitored using UV-Vis spectroscopy. As shown in Fig. 3b, the whole intensity of the UV-Vis curves increased with polymerization. Finally, the polymerization kinetics, represented by the peak intensity at 540 nm plotted against polymerization time, are shown in Fig. 3c. There was minimal change before 3 h, attributed to the induction period characteristic of RAFT PISA polymerization. After this period, rapid growth was observed, consistent with the optical images. To further illustrate the growth process, DLS measurements were also conducted during these same stages. As shown in Fig. S9, the nanoparticle diameters gradually increased from 100 nm to 290 nm, correlating well with the growth stages observed in both TEM images and UV-Vis spectra.

**Fig. 4:**
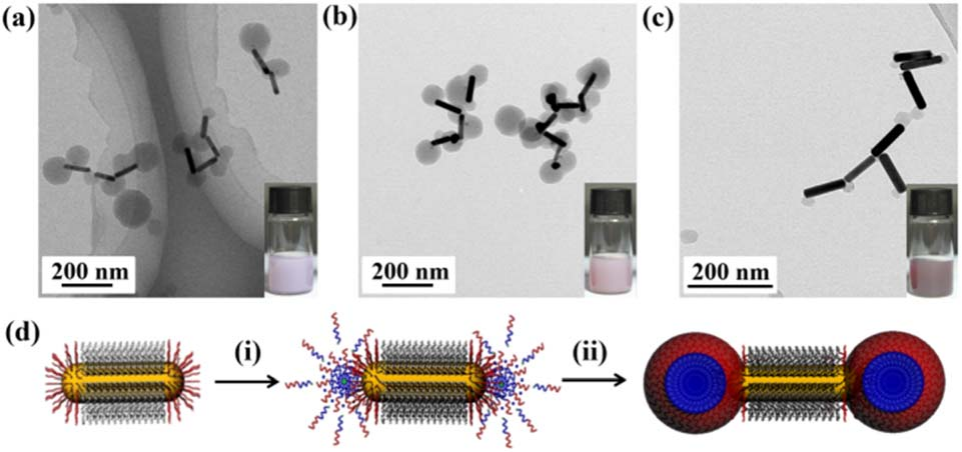
(a–c) TEM images and optical photographs obtained with three different recipes. (d) Schematic of the kinetic growth of dumbbell AuNRs@BCP *via* the UV light-initiated RAFT PISA method.

Based on the results presented above, we can infer that using a smaller quantity of AuNRs@end-P4VP seeds will lead to a lower yield of the dumbbell AuNRs@BCP nanoparticles. Additionally, the size of the polymer component can be controlled by adjusting the concentration of AuNRs@end-P4VP seeds. Consequently, three samples were designed and synthesized, with detailed reaction conditions provided in Table S2. When the minimum amount of AuNRs@end-P4VP was utilized, the resulting dispersion exhibited a pink-white turbidity. The TEM results shown in Fig. 4a indicated the presence of both dumbbell AuNRs@BCP and blank P4VP-*b*-PS micelles. In addition, the size of the BCP at the ends of AuNRs was generally larger than that of the blank micelles, which is attributed to a faster growth rate during the polymerization.^36^ If the homogeneous micelle nucleation growth pattern is eliminated, only a heterogeneous nucleation hybrid growth pattern exists. The optical image in Fig. 4b displayed a lighter purplish-red turbidity, and the TEM results showed that the AuNRs@BCP exhibited a relatively uniform size distribution with a yield of 100%. By further increasing the amount of AuNRs@end-P4VP, the resulting color shifted to a deeper purplish-red turbidity, and the TEM results indicated the presence of smaller-sized AuNRs@BCP (Fig. 4c). The UV-Vis absorption spectra of different nanoparticles in Fig. S10 showed that the smaller dumbbell AuNRs@BCP nanoparticles had two obvious SPR peaks. Based on the above experiments, we propose that the growth mechanism of dumbbell nanoparticles primarily consists of two stages (Fig. 4d): (i) homogeneous polymerization and loop micelle formation on the surface of AuNRs@end-P4VP; and (ii) heterogeneous polymerization in the micelle core, leading to the growth of larger structures.

**Fig. 5:**
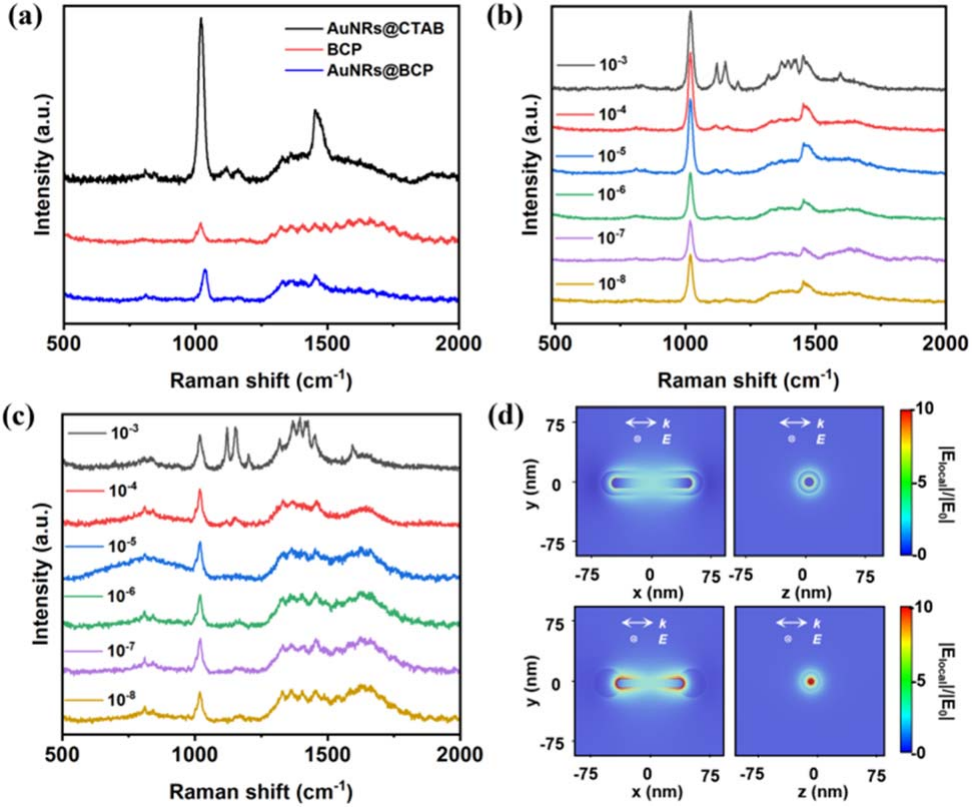
(a) Raman spectra of AuNRs@CTAB, AuNRs@BCP, and BCP dispersions. (b) SERS spectra of a mixture of core–shell AuNRs@BCP and MO with different concentrations. (c) SERS spectra of a mixture of dumbbell AuNRs@BCP and MO with different concentrations. (d) Numerically simulated electric field amplitude for the core–shell AuNRs@BCP and dumbbell AuNRs@BCP nanoparticles.

Due to this unique structure and composition, Raman spectra and SERS measurements with or without the addition of the MO molecule, were conducted in dilute dispersion.^49^ First, the Raman spectra of AuNRs, AuNRs@BCP, and BCP alone were characterized under a 785 nm laser. As shown in Fig. 5a, both AuNRs@BCP and AuNRs@CTAB exhibited two distinct Raman peaks at 1021.8 cm^−1^ and 1453.5 cm^−1^. Next, the shape-dependent SERS characteristics of different AuNR-based nanoparticles were studied. The SERS measurements were conducted after 30 min for MO adsorption equilibrium on the nanoparticle surfaces. The results in Fig. 5b showed that the core–shell AuNRs@BCP with different MO species could only detect concentrations as low as 10^−3^ M. The Raman peaks of MO at 1391 cm^−1^ and 1418 cm^−1^ are associated with the N=N stretching vibration; peaks around 1312 cm^−1^, 1445 cm^−1^, and 1591 cm^−1^ correspond to the C–C stretching vibrations; peaks at 1117 cm^−1^ and 1195 cm^−1^ are attributed to the phenyl-nitrogen (Ph-N) stretching vibration; and the peak at 923 cm^−1^ corresponds to the bending modes of C–H and C–C bonds.^50^ Surprisingly, the signal intensity of MO with dumbbell AuNRs@BCP in Fig. 5c could detect concentrations lowest to 10^−8^ M, whereas the SERS spectra signal intensity of MO with AuNRs@CTAB (Fig. S11) was only detectable down to 10^−5^ M. To better understand these shape-dependent SERS characteristics, we performed electric field simulations to calculate the electric field distributions around laser-illuminated AuNRs, dumbbell AuNRs@BCP, and core–shell AuNRs@BCP, which were used for constructing 3D models based on the experimental TEM data. The numerically simulated electric field amplitudes around the AuNRs, dumbbell AuNRs@BCP, and core–shell AuNRs@BCP are presented in Fig. 5d and S12, respectively. The results indicate that the maximum field enhancement appears at the two ends of the dumbbell AuNRs@BCP. Due to the plasmonic hotspots formed between AuNRs and BCP domains, the dumbbell AuNRs@BCP are ideal for generating strong and reproducible SERS signals.

## Conclusions

In summary, for the first time, novel dumbbell AuNRs@P4VP-*b*-PS nanoparticles were successfully prepared by the UV photoinitiated RAFT PISA method. A key factor in this process was the incorporation of AuNRs@end-P4VP in the system. The experimental results demonstrated that P4VP can induce styrene polymerization, growing symmetrically on both ends of AuNRs@end-P4VP to form dumbbell AuNRs@P4VP-*b*-PS nanoparticles *in situ*. The size of the nanoparticles can be adjusted by controlling the reaction time and the concentration of AuNRs@end-P4VP. Finally, SERS experiments and electric field simulations clearly showed that the dumbbell AuNRs@BCP produced strong SERS signals for MO molecules in dispersion. Compared to traditional self-assembly methods (Table S3), this study lays a solid theoretical foundation for the preparation and application of complex functional block copolymer/inorganic nanoparticle hybrids.

## Conflicts of interest

There are no conflicts to declare.

## Supplementary Material

RA-016-D5RA06140G-s001

## Data Availability

The data supporting this article have been included as part of the supplementary information (SI). Supplementary information: details of the experimental procedure and analytical data. See DOI: https://doi.org/10.1039/d5ra06140g.
