# Supplementary material for: Synthesis of the dumbbell AuNRs@BCP nanoparticles via UV light-initiated RAFT polymerization-induced self-assembly
Source: RSC Adv. 2026 Jan 29;16(7):6210–6. doi: 10.1039/d5ra06140g (PMC12854663; doi:10.1039/d5ra06140g)
Supplement: RA-016-D5RA06140G-s001 [file RA-016-D5RA06140G-s001.pdf]

Supplementary Information (SI) for RSC Advances.
This journal is © The Royal Society of Chemistry 2026

Supporting Information for

Synthesis of dumbbell AuNRs@BCP nanoparticles via UV light-initiated RAFT polymerization-induced self-assembly

Jian Wang,[a] Feng Xu,[a] Zhenzhong Liu,*[b] Linli Xu,[c] Mingyue Liu,*[c] Ye Deng,[a] Yue Sun,[b] Yanjing Zhang,[c] Dan-hua Wang[a] and Youju Huang*[d]

a. Chemical Pharmaceutical Research Institute, College of Medicine and Pharmaceutical Engineering, Taizhou Vocational and Technical College, Taizhou 318000, China

b. Taizhou Key Laboratory of Medical Devices and Advanced Materials, Taizhou Institute of Zhejiang University, Taizhou 318000, China

c. School of Pharmaceutical and Chemical Engineering, Taizhou University, Taizhou 318000, China

d. College of Materials, Chemistry and Chemical Engineering, Hangzhou Normal University, Hangzhou 311121, China.

E-*mail: zzliu@zju.edu.cn; liumingyue0820@126.com; yjhuang@hznu.edu.cn*

Data analysis:

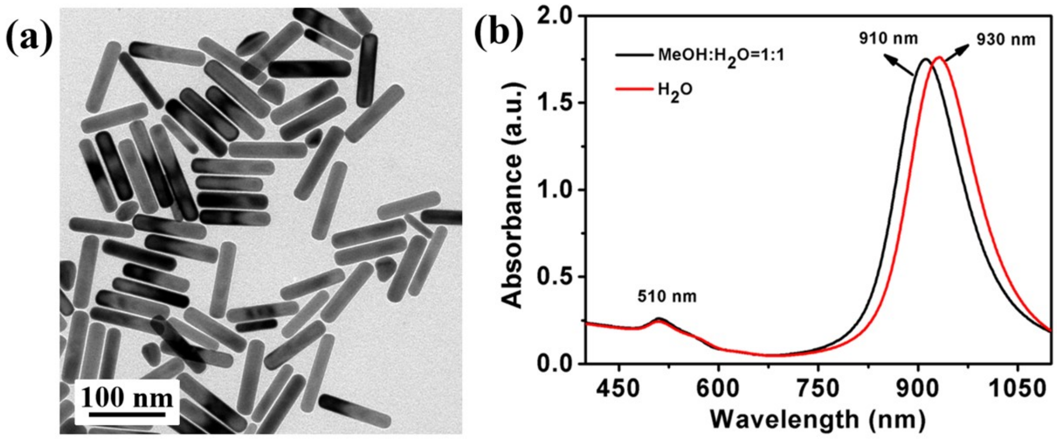

Figure S1. (a) The TEM of AuNRs, (b) the UV-Vis spectra of AuNRs in H_2O and the mixture of methonal:H_2O=1:1.

Table S1. Experimental fomula directly using AuNRs@CTAB as seeds by UV-light initiated RAFT PISA method.

No.	AuNRs@CTAB[a]	P4VP-CTA	St	AIBN	methanol	H_2O
1	500 μL	4 mg	0.08 mL	0.02 mg	3.75 mL	0.5 mL

[a] The AuNRs@CTAB is concentrated in H_2O and stored at 5 °C before use. Experimental condition: UV light intentsity: I_{365} = 2.50 mW cm^{-2}, irradiation time: 18 h, 25 °C.

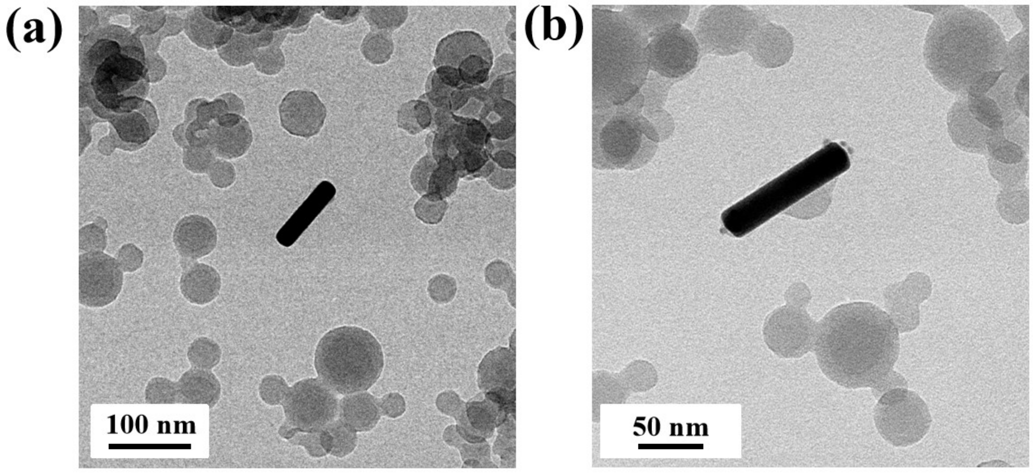

Figure S2. The TEM images of the mixture of AuNRs and micelles by directly using AuNRs without modification under UV ligh-inithiated RAFT PISA process.

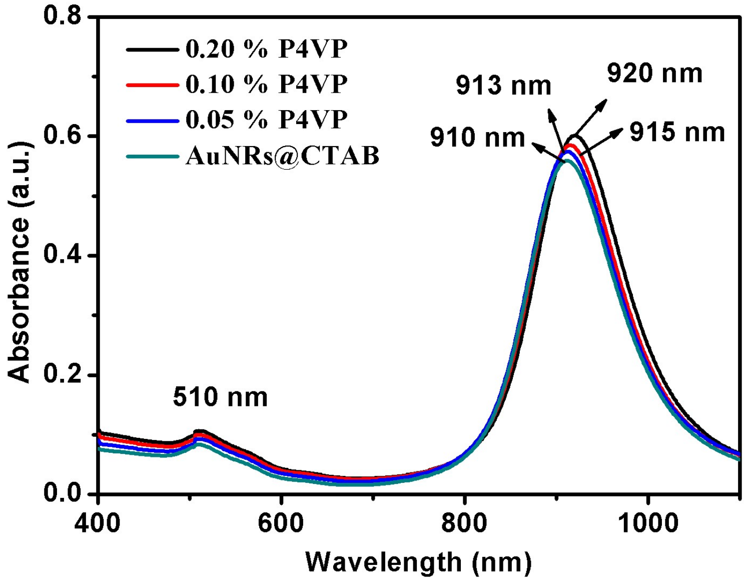

Figure S3. The UV-Vis spectra of AuNRs@P4VP modified with different contents of P4VP-CTA in methonal:H_2O = 1:1.

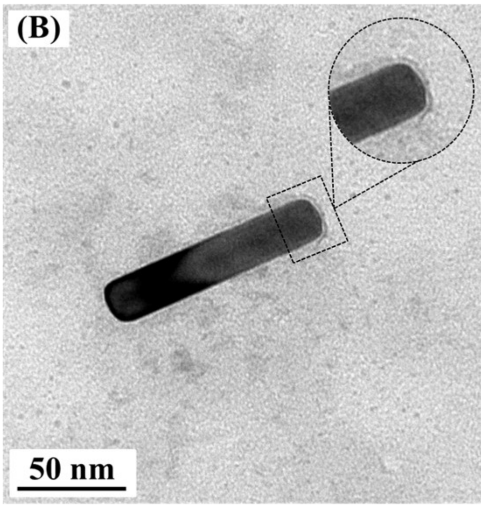

Figure S4. TEM images of modified AuNRs@all-P4VP.

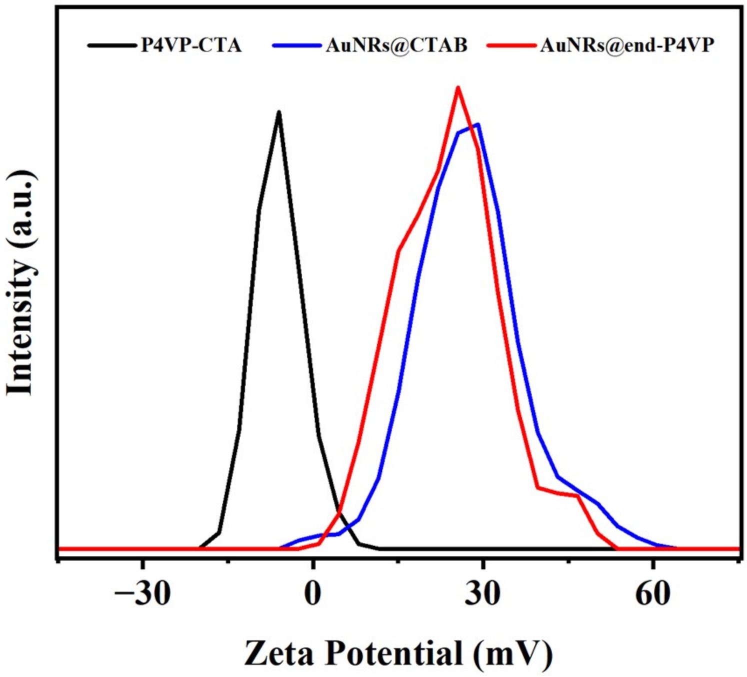

Fig. S5. The comparison zeta potential values of P4VP-CTA, AuNRs@CTAB, and AuNRs@end-P4VP in water.

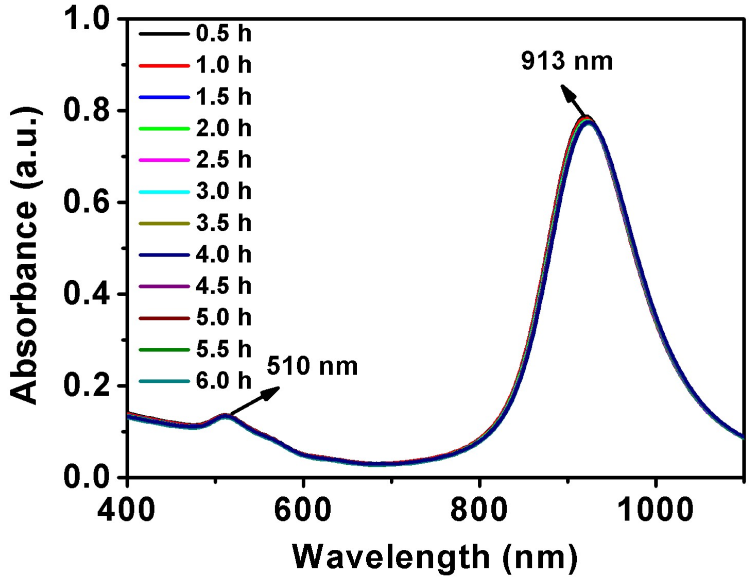

Figure S6. The UV-Vis spectra of AuNRs@end-P4VP in methonal : H_2O = 1:1 under UV light irridiation for 6 h.

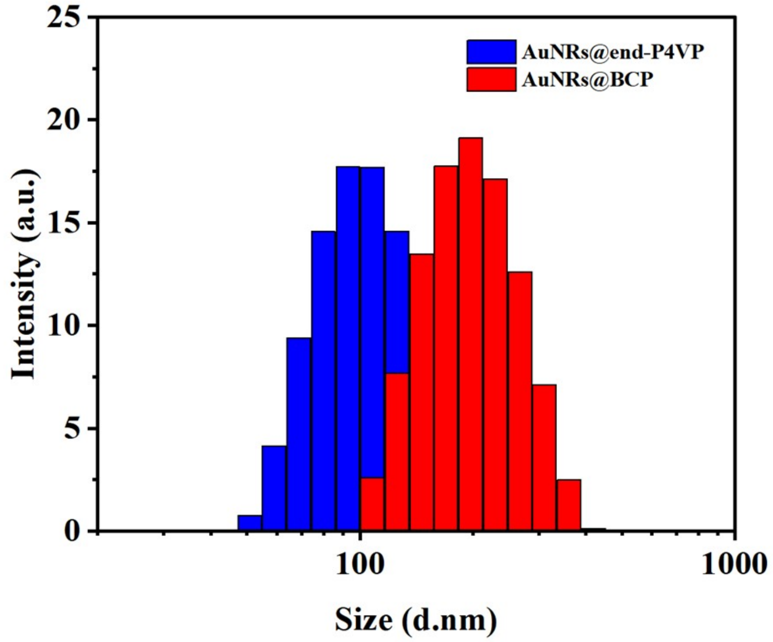

Fig. S7. The comparison diameter distribution values of AuNRs@end-P4VP, and the dumbbell AuNRs@BCP in water.

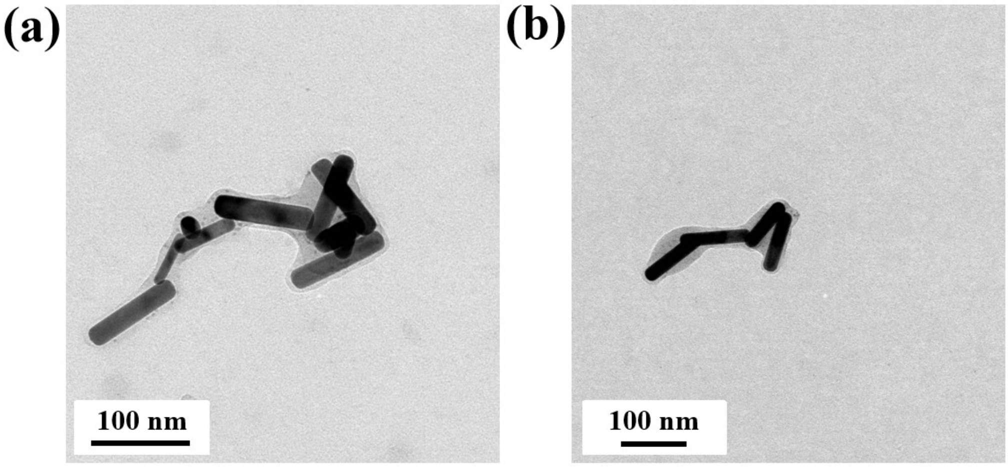

Figure S8. TEM images of core-shell AuNRs@BCP nanoparticles by using AuNRs@all-P4VP under UV ligh-initiated RAFT PISA process.

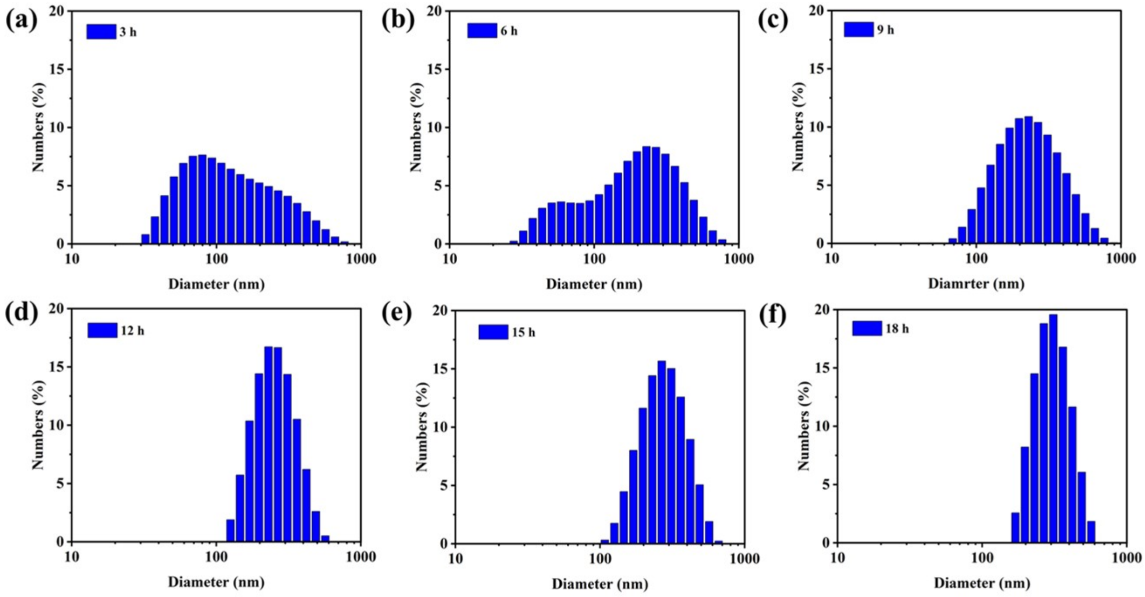

Fig. S9. The comparison diameter distribution changes which were monitored by DLS measurement via the UV light-initiated RAFT PISA method at different polymerization times: (a) 3h, (b) 6h, (c) 9h, (d) 12h, (e) 15h, and (f)18h.

Table S2. Experimental fomula using AuNRs@end-P4VP as seeds by UV-light initiated RAFT PISA method.

No.	AuNRs@ end-P4VP[a]	P4VP	St	AIBN	methanol	H_2O
1	500 μL	4 mg	0.08 mL	0.02 mg	3.75 mL	0.5 mL
2	750 μL	4 mg	0.08 mL	0.02 mg	3.75 mL	0.5 mL
3	1000 μL	4 mg	0.08 mL	0.02 mg	3.75 mL	0.5 mL

[a] The AuNRs@end-P4VP is concentrated in methanol and stored at 5 °C before use. Experimental condition: UV light intentsity: I_{365} = 2.50 mW cm^{-2}, irradiation time: 18 h, 25 °C.

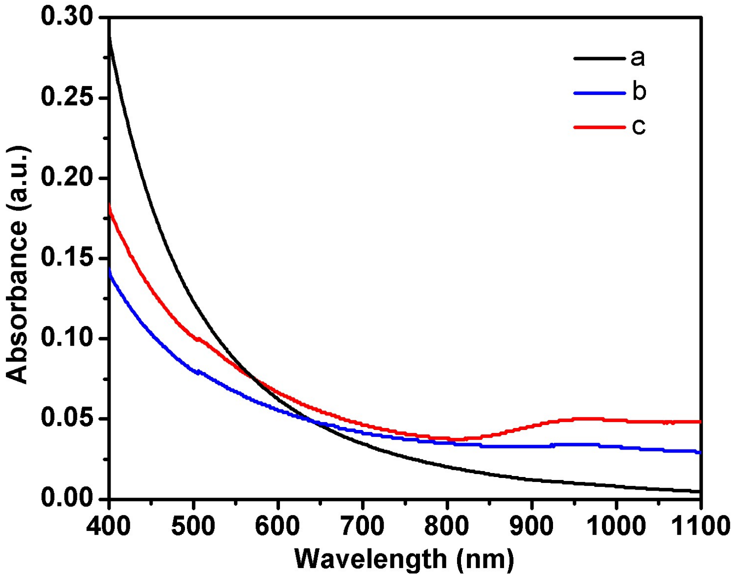

Figure S10. The UV-Vis absorption spectra of different nanoparticles prepared by UV light initiated RAFT PISA method: (a) P4VP-*b*-PS micelles and dumbbell AuNRs@P4VP-*b*-PS nanoparticles, (b) larger dumbbell AuNRs@P4VP-*b*-PS nanoparticles, (c) smaller dumbbell AuNRs@P4VP-*b*-PS nanoparticles.

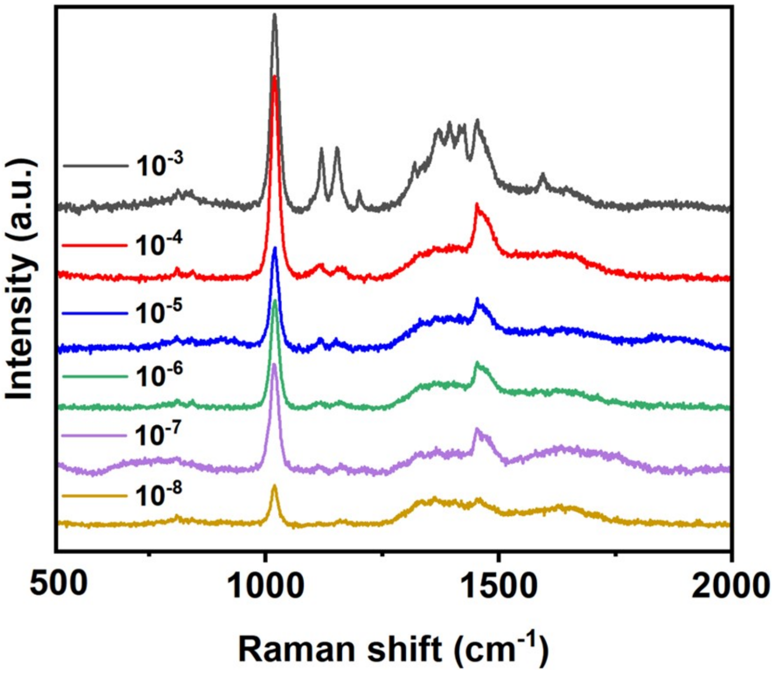

Fig. S11. SERS spectra of MO with different concentrations at 10^{-3}-10^{-8} M on core-shell AuNRs in dispersion.

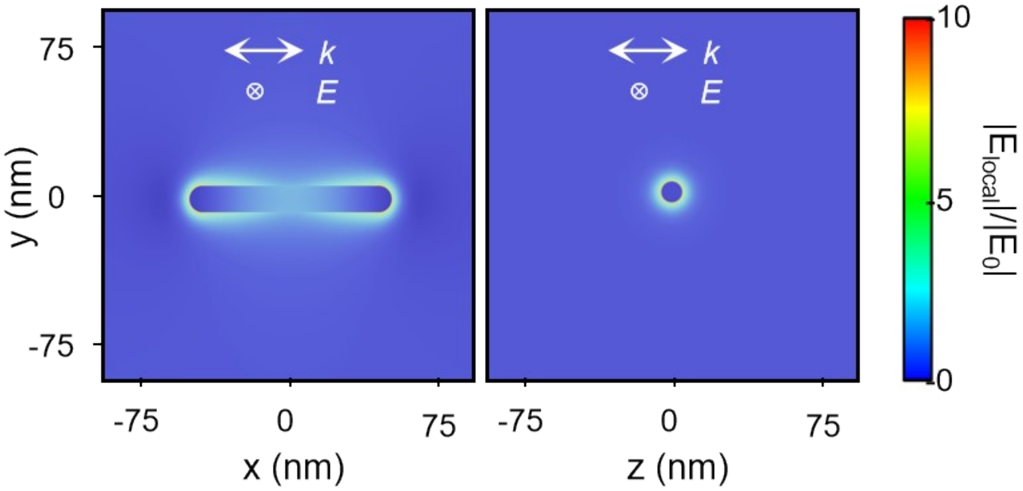

Fig. S12. The numerical simulated electric field amplitude for AuNRs.

Table S3. The comparative analysis of different self-assembly methods to prepare hybrid inorganic/block copolymers nanomaterials.

No.	Methods	Materials	Characteristics/Yields	Applications	References
1	Directed supramolecular assembly	P4VP-*b*-PS, AuNRs@PS	Cylindrical PS domain, dilute solutions	—	Ref 1
2	Solution self-Assembly	PS-*b*-PAA, gold (spheres, nanorods and nanostars)	Core-shell hybrid nanomaterials, dilute miscible mixture and heating	—	Ref 2
3	Heat-induced transformation	PS-*b*-PAA, AuNRs	different modes (contraction, bimodal contraction, dissociation, and winding), dilute solution mixture and heating	—	Ref 3
4	Heat polymerization-induced surface self-assembly	silica particles	fabrication of surface nanostructures, heat polymerization	—	Ref 4
5	UV light Polymerization-Induced Self-Assembly	P4VP-*b*-PS, AuNPs	Janus AuNPs@BCP, photopolymerization, high yields contents	—	Ref 5
6	UV light Polymerization-Induced Self-Assembly	PS-b-P2VP, AuNRs	Dumbbell AuNPs@BCP, tunable morphologies, photopolymerization	SERS	This work

Reference:

1. W. Li, P. Zhang, M. Dai, J. He, T. Babu, Y. Xu, J. Zhu, Macromolecules, 2013, 46, 2241-2248.

2. M. Grzelczak, A. Sanchez-Iglesias, L. Liz-Marzan, CrystEngComm, 2014, 16, 9425-9429.

3. Z. Wang, B. He, G. Xu, G. Wang, J. Wang, Y. Feng, H. Chen, Nat. Commun., 2018, 9, 563.

4. W. Hou, H. Wang, Y. Cui, Y. Liu, X. Ma, H. Zhao, Macromolecules, 2019, 52, 8404-8414.

5. Z. Liu, C. Wu, Y. Fu, X. Xu, J. Ying, J. Sheng, T. Chen, Nanoscale Adv., 2021, 3, 347-352.
